# Cervical ulcer caused by group B streptococcus with bacterial vaginosis: a case report

**DOI:** 10.1186/s12905-023-02665-w

**Published:** 2023-09-27

**Authors:** Yi Chen, Dan Wu

**Affiliations:** 1 Cervical Center of The International Peace Maternal and Child Health Hospital, School of Medicine, Shanghai Jiao Tong University, Shanghai, 200030 China; 2 Shanghai Key Laboratory of Embryo Original Diseases, Shanghai, 200030 China

**Keywords:** Cervical ulcer, *Streptococcus*, Bacterial vaginosis, Genital ulcer

## Abstract

The female genital ulcer is a manifestation of many diseases, which may vary depending on the etiology, disease duration, age, and host immunity. A middle-aged (40–50 years) woman had a 4-month history of vaginal bleeding. The results of syphilis, herpes, the cervical cancer, tuberculosis, and fungi or acute cervical inflammation caused by Chlamydia trachomatis and Mycoplasma hominis were negative through the blood test and the biopsy. Cervical discharge culture revealed positive for group B *Streptococcus* and bacterial vaginosis. The patient was treated with oral antibiotics for 7 days. One month later, repeat colposcopy revealed a smooth cervix and complete ulcer disappearance, while cervical discharge culture retested no group B *Streptococcus* and bacterial vaginosis. The patient was diagnosed with cervical ulcer. Complete medical history taking and bacterial culture of cervical discharge are important for identifying the etiology of the cervical ulcer and deciding the appropriate treatment for the disease.

## Introduction

Ulceration refers to the disruption of the continuity of the skin and/or mucous membrane and involves loss of the epidermis, dermis, and subcutaneous tissue [1]. The female genitalia can be easily infected with various organic diseases. The unique anatomical location of the genital area hinders women’s self-examination. Additionally, the warm and humid environment in the area results in various disease presentations. The female genital ulcer, a very common disease, is a manifestation of many diseases, which may vary depending on the etiology, disease duration, age, and host immunity, resulting in difficulties in diagnosis and unsatisfactory treatment results [2]. It is a type of tissue necrosis caused by cell damage due to intense local inflammation and ischemia. The World Health Organization (WHO) recommends that the patients should receive a comprehensive treatment for genital ulcers at the first visit, which would reduce the chance of infection with other diseases [3]. However, in many cases, genital ulcer could not be cured by empirical treatment. If treated as a venereal disease, it can lead to marital discord. In fact, many genital ulcer tissue injuries (necrosis) are caused by not only sexually transmitted diseases (STDs), but also ischemia, autoimmune diseases, or even stimulation phenomena [4]. Cervical ulcers are typically caused by cervical cancer and STDs, however, cervical ulcers can also develop in patients with Behcet’s syndrome or tuberculosis. Current study reported a rare case of cervical ulcer caused by group B *Streptococcus* (GBS) with bacterial vaginosis (BV).

## Case presentation

A middle-aged (40–50 years) woman with a 4-month history of vaginal bleeding was admitted to the Cervical Center of the International Peace Maternity & Child Health Hospital of China Welfare Institute. Four months ago, the patient underwent colposcopy guided multipoint biopsy and endocervical curettage in another hospital, and was diagnosed with chronic cervicitis. After the biopsy, the patient had vaginal bleeding, which was diagnosed as cervical ulcer and cervical cancer was rules out. Vaginal discharges were negative for acid-fast bacilli. Despite 4 months of treatment with oral tranexamic acid tablets and local vaginal interferon suppositories, the symptoms did not improve. The patient had no history of recurrent oral ulcers. Physical examination showed no oral mucosa and skin ulcerations. There were no ocular lesions and no visual deterioration on ophthalmological examination. Speculum examination showed no vulvar ulcerations and smooth vaginal walls with intact mucous membrane. She had heavy bloody vaginal discharge but was not foul smelling.

**Fig. 1:**
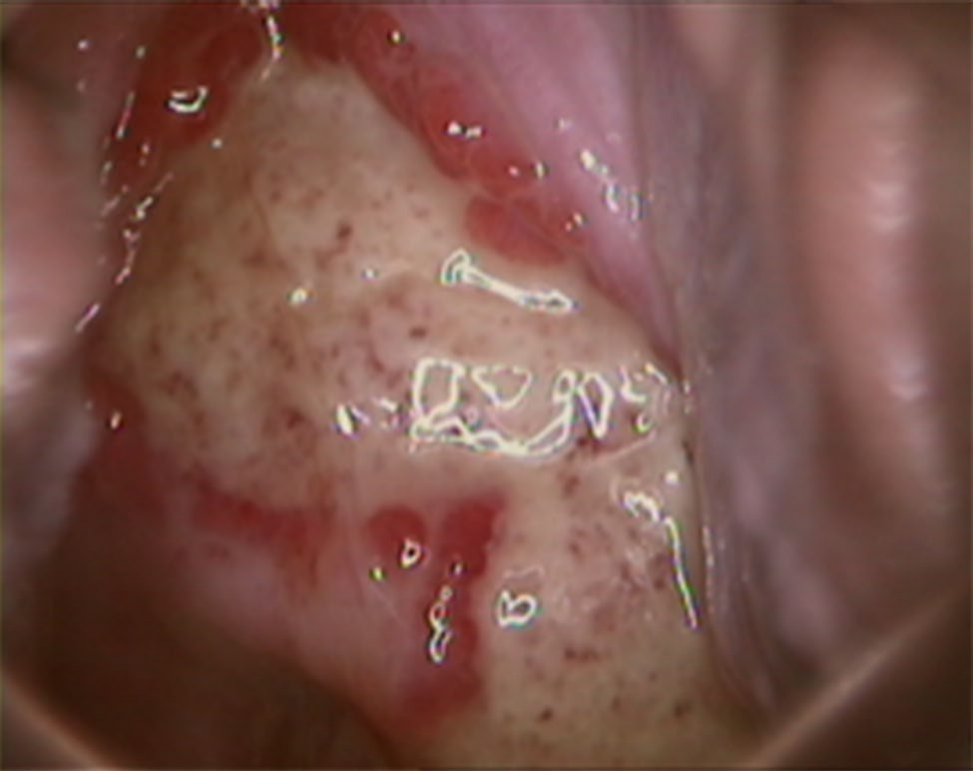
Cervical ulcer and marginal tissue exposed under colposcopy after removal of cervical surface secretions before treatment

**Fig. 2:**
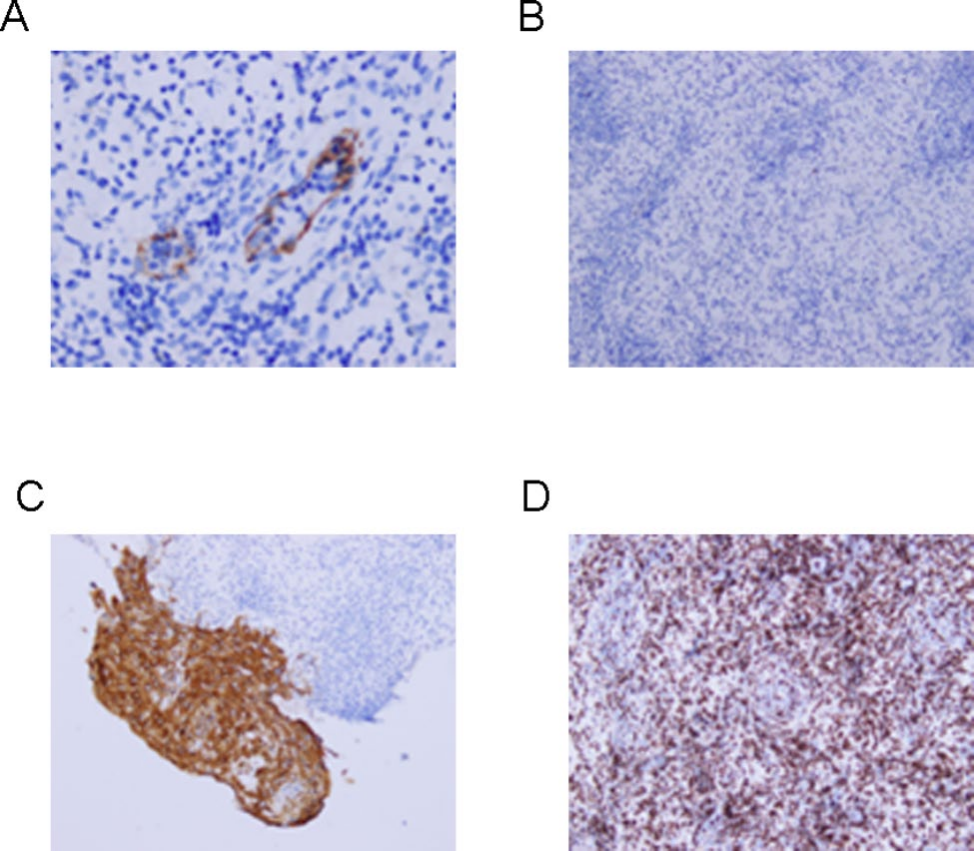
The pathological results of the biopsy. **(A)** CD31(+); **(B)** Desmin (+); **(C)** EMA (+); **(D)** LCA (+)

We performed a cervical liquid-based thin layer cell test that showed no cervical intraepithelial neoplasia. She was negative for syphilis and the immunoglobulin M of herpes simplex virus according to the blood test. Cervical discharge was positive for BV but negative for the deoxyribonucleic acid of type II herpes simplex virus, high risk human papilloma virus, and human papilloma virus 6/11. Cervical discharge culture was positive for GBS, but negative for fungi, *Ureaplasma urealyticum, Mycoplasma hominis*, and *Chlamydia trachomatis* antigen. To further rule out cervical cancer, colposcopy and biopsy were performed on February 27, 2018 (Fig. 1). The pathological results of the biopsy were as follows: presence of inflammatory granulation tissue, immunohistochemical cluster of differentiation 31 (CD31) (+), Desmin (+), epithelial membranous antigen (EMA) (+), and lymphocytotoxic antibody (LCA) (+) (Fig. 2).

**Fig. 3:**
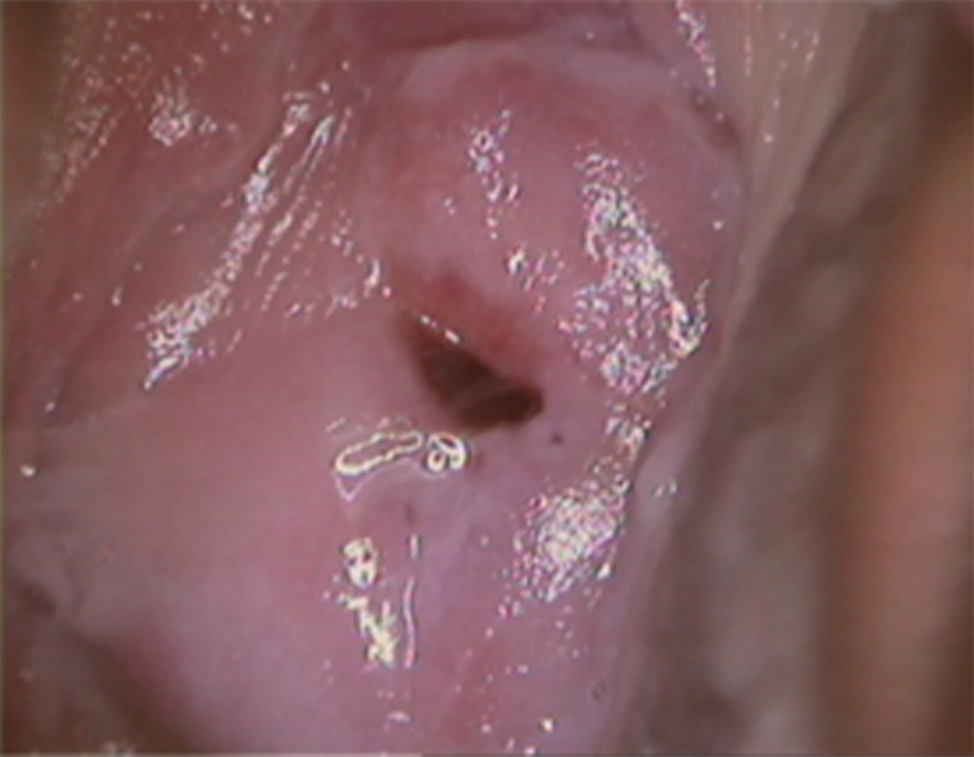
After treatment, the cervix was smooth under colposcopy and the ulcer disappeared completely

Based on the patient’s disease history, diagnosis, and treatment, cervical ulcer caused by GBS with BV was considered. The patient was treated with 400 mg metronidazole twice daily + 500 mg cefradine four times daily for 7 days. One month later, the repeat colposcopy revealed a smooth cervix and complete ulcer disappearance (Fig. 3), meanwhile, the cervical discharge culture showed no group B Streptococcus and bacterial vaginosis.

## Discussion

Cervical ulcers may be caused by non-STDs, such as cervical cancer, serious vulvovaginal candidiasis, cervical tuberculosis, and Behcet’s syndrome. However, STDs, such as syphilis chancre and HSV2 infection, may also cause cervical ulcers. In this case, cervical biopsy under colposcopy showed the presence of inflammatory granulation tissues, thereby ruling out cervical ulcer caused by cervical cancer, syphilis, tuberculosis, herpes, and fungi or acute cervical inflammation caused by *Chlamydia trachomatis* and *Mycoplasma hominis*. Given that the cervical ulcer of this patient is rarely encountered in clinics, the possibility of Behcet’s syndrome should be considered in patients presenting these signs and symptoms.

Behcet’s syndrome is characterized by multiple tissue and organ damage caused by autoimmune disorders, with repeated attacks and remission. Oral cavity, eyes, genitals, and skin are the most frequent injury sites There is no specific serological and pathological characteristics. The diagnosis is mainly based on clinical symptoms and signs [5]. About 57-93% of patients with Behcet’s syndrome have genital ulcers, mainly in the vulva, but the cervix can also be affected. The lesion is similar with oral ulcers [6]. The diagnostic criteria of Behcet’s syndrome requires the presence of oral ulcerations plus any two of the following: genital ulceration, typical defined eye or skin lesions, or a positive pathergy test [7]. Our patient had clear vision, no visual deterioration, no history of recurrent oral ulcer, and no oral and skin ulcerations upon physical examination, therefore, Behcet’s syndrome could be ruled out. The patient’s cervical biopsy suggested that inflammatory cells in the tissues infiltrate and grow into new capillaries without cancerous cells. Vaginal discharge culture was positive for BV and had growth of GBS, which was the main causative bacteria of the cervical ulcer.

GBS is facultative anaerobic bacteria and is an opportunistic pathogen for pregnant women, newborns, and the elderly [8]. GBS exists in the vagina or lower digestive tract in 10-40% of women [9]. GBS infection can lead to intrauterine infection, abortion, premature delivery, premature rupture of membranes, and other adverse pregnancy outcomes, which cause severe adverse consequences, such as neonatal pneumonia, meningitis, and septicemia. Adult men and women can also have severe GBS infection, such as urinary tract infection, pneumonia, or soft tissue infection, which are the most common diseases in adults [10]. With the widespread use of antibiotics, research focusing on the drug resistance to GBS and drug sensitivity test in vitro in recent years showed that GBS had a high drug resistance to erythromycin and clindamycin [11]. Antibacterial drugs with no or low drug resistance included cephalosporins, penicillin, vancomycin, and so on [12]. Given that vancomycin has certain hepatorenal toxicity to the body, it is not recommended as the first drug choice. Although some cases with *Streptococcus* infection had drug resistance to penicillin, their drug resistance was relatively low. Therefore, penicillin can be used as the first choice in the treatment of GBS, and cephalosporin antibiotics can be used for patients allergic to penicillin. In our study, this patient had also BV. Metronidazole is the preferred drug for treating BV [13, 14]. Accordingly, the patient was orally administered 500-mg cefradine four times daily + 400-mg metronidazole twice daily for 7 days, and the cervical ulcer was successfully treated.

In conclusion, based on the screening of cervical cancer and STDs, cervical ulcers caused by non-STDs should be paid attention. Complete medical history and bacterial culture of cervical secretions can help identify the etiology of the disease and decide the appropriate targeted treatment.

## Data Availability

The datasets during and/or analyzed during the current study are available from the corresponding author on reasonable request.

## Abbreviations

WHO: the World Health Organization
STDs: sexually transmitted diseases
GBS: group B *Streptococcus*
BV: bacterial vaginosis
CD31: cluster of differentiation
EMA: epithelial membranous antigen
LCA: lymphocytotoxic antibody
